# Hematopoietic Stem Cell Transplantation for the Treatment of Autoimmune Neurological Diseases: An Update

**DOI:** 10.3390/bioengineering10020176

**Published:** 2023-01-29

**Authors:** Alice Mariottini, Giovanni Bulgarini, Sara Cornacchini, Valentina Damato, Riccardo Saccardi, Luca Massacesi

**Affiliations:** 1 Department of Neurosciences, Drug and Child Health, University of Florence, 50139 Florence, Italy; 2 Department of Neurology 2, Careggi University Hospital, 50134 Florence, Italy; 3 Cell Therapy and Transfusion Medicine Unit, Careggi University Hospital, 50134 Florence, Italy

**Keywords:** hematopoietic stem cell transplantation, autoimmune diseases of the nervous system, neuromyelitis optica, myasthenia gravis, polyneuropathy, myopathies, Behcet, vasculitis, nervous system diseases

## Abstract

Over the last two decades, haematopoietic stem cell transplantation (HSCT) has been explored as a potential therapeutic strategy for autoimmune diseases refractory to conventional treatments, including neurological disorders. Although both autologous (AHSCT) and allogeneic HSCT (allo-HSCT) were investigated, AHSCT was preferentially developed due to a more favourable safety profile compared to allo-HSCT. Multiple sclerosis (MS) represents the most frequent neurological indication for AHSCT, but increasing evidence on the potential effectiveness of transplant in other autoimmune neurological diseases is emerging, although with a risk-benefit ratio overall more uncertain than in MS. In the present work, the rationale for the use of HSCT in neurological diseases and the experimental models that prompted its clinical application will be briefly covered. Case series and prospective studies exploring the use of HSCT in autoimmune diseases other than MS will be discussed, covering both frequent and rare neurological disorders such as myasthenia gravis, myopathies, and stiff-person syndrome. Finally, an updated summary of ongoing and future studies focusing on this issue will be provided.

## 1. Introduction

Over the last two decades, haematopoietic stem cell transplantation (HSCT) has been explored as a potential treatment for aggressive autoimmune diseases, including autoimmune neurological disorders [1]. Both autologous (AHSCT) and allogeneic HSCT (allo-HSCT) have been investigated, but AHSCT was preferentially developed due to its more favourable safety profile compared to allo-HSCT. Multiple sclerosis (MS) represents the most frequent autoimmune neurological indication for AHSCT, with 1998 patients treated up to August 2022 and reported to the Registry of the European Blood and Marrow Transplantation Society (EBMT, www.ebmt.org, accessed on 21 December 2022) [2]. Growing evidence on the efficacy and safety of AHSCT in MS, including the results of a randomised control study [3], prompted the endorsement of AHSCT as a “standard of care” for highly active relapsing MS refractory to conventional treatments by the EBMT and American Society for Blood and Marrow Transplantation guidelines [1,4]. In addition to MS, other autoimmune neurological diseases have been successfully treated with HSCT, although the number of treated cases is lower and the risk-benefit ratio is more uncertain than in MS. The vast majority of the patients have received autologous transplants, while less than 20 allo-HSCTs for neurological autoimmune disorders are reported in the registries for the period 1997–2014 [5], and autoimmune diseases represented the indication for 0.1% of allo-HSCT and 1.3% of AHSCT in the 2020 EBMT registry report [6]. Up to 20 December 2022, 131 AHSCT were reported in the EBMT registry for the treatment of neurological autoimmune diseases in the adult setting: chronic inflammatory demyelinating polyneuropathy (CIDP) was the most frequent indication (n = 62), followed by neuromyelitis optica (NMO; n = 21), myasthenia gravis (MG) and stiff-person syndrome (SPS) (11 cases each), and encephalitis (n = 5); the remaining 21 AHSCT were performed for other autoimmune neurological diseases [7]. Nine allo-HSCTs were also reported in this setting: two for CIDP, three for NMO, one for encephalitis, and three for other neurological autoimmune disorders [7].

The present descriptive review aimed at reviewing the available evidence on the use of HSCT in autoimmune neurological diseases other than MS, providing an updated summary of the latest findings in recent prospective studies. Experimental models that prompted the clinical application of HSCT will be briefly covered, and ongoing and future studies on HSCT in neurological diseases will be summarised.

## 2. Materials and Methods

### 2.1. Article Search

Research articles and conference abstracts reporting data on the effectiveness and safety of HSCT as a treatment of autoimmune neurological diseases other than MS were searched in the databases PubMed, Embase, and clinicaltrials.gov. The terms “hematopoietic stem cell transplantation” AND each of the following were singularly adopted: stiff-person syndrome, neuromyelitis optica/ neuromyelitis optica spectrum disorder, myasthenia gravis, chronic inflammatory demyelinating polyneuropathy, neuropathy, vasculitis of the central nervous system (CNS), encephalitis, myopathy, and neurological autoimmune diseases (excluding MS). Articles not written in the English language and not related to humans were excluded. Articles were independently searched by two authors. After the exclusion of duplicates, the authors first screened the studies for eligibility on the basis of their titles and abstracts. Discrepancies were discussed by the authors and resolved after re-examination of the source studies.

### 2.2. Statistical Analysis

Baseline characteristics of the patient population and outcome variables were reported according to the summary statistic used in each study.

Where not available, the summary statistic was extrapolated from raw individual patient data (if available), providing mean values (range) for continuous variables and numbers (frequency) for dichotomous variables.

## 3. Haematopoietic Stem Cell Transplantation in Autoimmune Diseases

### 3.1. Transplant Procedure

**Figure 1:**
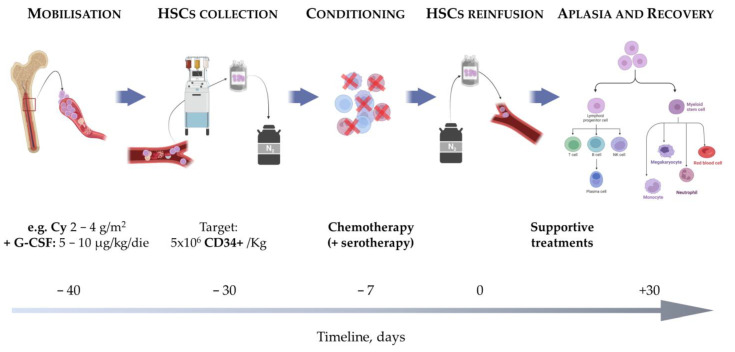
Haematopoietic stem cell transplantation (HSCT) procedure. HSCT is a multistep procedure encompassing: mobilisation of the haematopoietic stem cells (HSCs), usually obtained with cyclophosphamide + granulocyte-colony stimulating factor, the latter administered until harvest collection. HSCs are then collected through leukapheresis and cryopreserved until transplant, which usually occurs after 30–40 days. During the conditioning, chemotherapy (with or without serotherapy) is administered to induce the ablation of the immune system. HSCs are then reinfused on day 0 of transplant, promoting the gradual recovery from the aplastic phase with reconstitution of the immune system. Abbreviations: Cy, cyclophosphamide; HSCs, haematopoietic stem cells; G-CSF, granulocyte-colony stimulating factor. Created with BioRender.com.

HSCT is a procedure consisting of four main steps that induces the ablation of the immune system and promotes its replacement with a “newly”-generated one derived from the differentiation and maturation of the haematopoietic stem cells (HSCs) (Figure 1).

HSCs are previously collected from the individual himself in an autologous transplant or from a healthy donor in an allogeneic transplant. HSCs are normally resident within the bone marrow and can be mobilised into the blood by the administration of granulocyte colony-stimulating factor (G-CSF, 5–10 µg/Kg day), usually preceded by chemotherapy to strengthen the mobilisation and prevent disease flare (e.g., cyclophosphamide—Cy, recommended dose 2 g/m^2^ body surface area, BSA) [1]. HSCs are then collected through leukapheresis (the optimal target is 5 × 10^6^ CD34^+^ cells/kg, with 2 × 10^6^/kg as a minimum safety threshold) and cryopreserved until transplant, usually scheduled 30–40 days after the mobilisation. The ablation of the immune system is induced by the administration of high-dose chemotherapy (alone or associated with serotherapy) during the conditioning phase, performed as an inpatient procedure.

**Figure 2:**
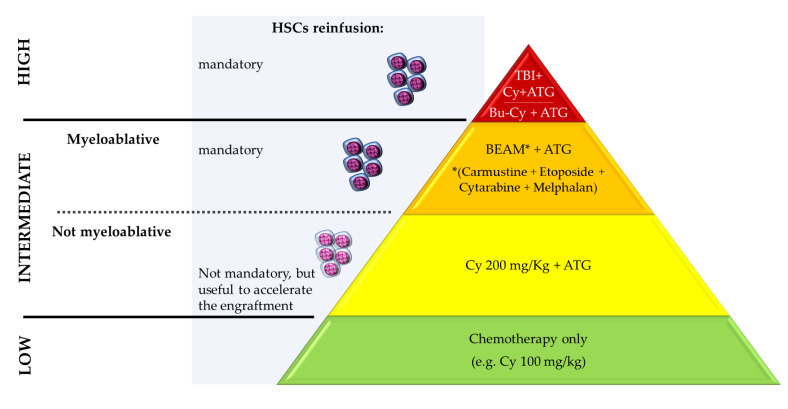
EBMT classification of conditioning regimens for haematopoietic stem cell transplantation, according to the intensity of immunoablation induced (high-, intermediate-, or low-intensity). The reinfusion of the haematopoietic stem cells (HSCs) is mandatory to promote the recovery from the aplastic phase after high and intermediate myeloablative regimens; it is recommended to accelerate the recovery after intermediate non-myeloablative and low-intensity regimens. Examples of the most commonly used chemotherapy drugs are reported. Abbreviations: ATG, anti-thymocyte globulin; Bu, busulphan; Cy, cyclophosphamide; HSCs, haematopoietic stem cells; TBI, total body irradiation.

Different conditioning regimens are available and classified by the EMBT guidelines according to the extent of their “myeloablative potential” into high-, intermediate-, or low-intensity regimens [1] (Figure 2).

The administration of the conditioning regimen is followed by HSCs reinfusion (considered as day 0 of the transplant); the graft may be reinfused unmanipulated or after ex-vivo purging by positive selection of CD34^+^ cells in order to deplete immune cells. T cell depletion may also be performed in vivo through the administration of anti-thymocyte globulin (ATG) or monoclonal antibodies (MoAbs), such as rituximab or alemtuzumab, together with the conditioning regimen. During the haematological recovery, patients receive supportive treatments and antimicrobial therapy for the prevention of common complications. In patients receiving allo-HSCT, the prolonged administration of immunosuppressive treatments is required to prevent graft-versus-host disease.

### 3.2. Experimental Models

The first animal models of bone marrow transplantation (BMT) in autoimmune disease were developed by Ikehara and colleagues in 1985 using murine strains with spontaneous systemic lupus erythematosus (SLE)-like disease [8] (Table 1). They were able to show that irradiation followed by allogeneic BMT from healthy mouse donors could prevent the development of the autoimmune disease when treating mice in the pre-clinical stage and treat the disease in those who had already developed the typical clinical and anatomopathological picture. Two different animal models were then developed: adjuvant arthritis (AA) in rats as a model for rheumatoid arthritis and experimental allergic encephalomyelitis (EAE) in rats, mice, and guinea pigs as a model for MS. In these models, high-dose total body irradiation (TBI) was used as a conditioning regimen to induce haemato-lymphatic ablation, followed by immunologic reconstitution with BMT; the graft could be allogeneic, syngeneic, or autologous [9,10,11,12,13].

Moreover, an animal model of MG in rats was developed by Pestronk’s group to investigate the therapeutic effect of autologous BMT. Even if the end-point was not muscular weakness, since clinical symptoms were absent or minimal in this model, the group showed that BMT, preceded by Cy and a sublethal dose of TBI, caused a rapid and sustained fall of anti-acetylcholine receptor (AChR) antibodies [14].

All these animal models (summarised in Table 1) proved that BMT was highly effective in autoimmune diseases, suggesting the exploration of transplant in the treatment of selected patients with severe autoimmune disease.

### 3.3. Mechanism(s) of Action

Data on immunological recovery suggestive of a reshaping of the immune system following AHSCT were provided in studies on MS patients, as well as in patients affected by SLE, juvenile arthritis, or systemic sclerosis [15,16,17].

Extensive renovation of the T cell receptor (TCR) repertoire with the renewal of the clonal specificities in both cerebrospinal fluid (CSF) and serum was demonstrated [18,19,20], as were broad changes in T cell subsets with depletion of pro-inflammatory phenotypes and promotion of an anti-inflammatory environment, associated with deep modification of gene expression profiles [21,22]. Importantly, MS patients who failed to respond to treatment had less diversity in their TCR repertoire [20]. Moreover, in patients with juvenile idiopathic arthritis and juvenile dermatomyositis, AHSCT induces functional renewal of regulatory T cells (Tregs) as well as a strong Treg TCR diversification [23,24].

Few data are available on the humoral changes after AHSCT. In patients with SLE, clinical remission after AHSCT was marked by the depletion of pathogenic anti-double-stranded DNA (dsDNA) antibodies and protective antibodies in serum and a normalisation of the previously disturbed B cell homeostasis with recovery of the naïve B cell compartment within 1 year after AHSCT [15]. Similarly, the negativisation of anti-aquaporin 4 (AQP4) antibodies was reported in a proportion of NMO spectrum disorder (NMOSD) patients after AHSCT, and this was associated with successful clinical outcomes [25].

Regarding the repopulation kinetics of immune cell subsets in the periphery post-AHSCT, data from MS patients studied 2 years post-therapy, showed that innate cells appear first, followed by abundant naïve B cells and late effector subsets of CD4 and CD8 T cells, together with transient increases in proportions of Tregs [26]. In general, compared to other immune cell types, B cell reconstitution occurs relatively late after HSCT. The first emerging transitional B cells can be detected in peripheral blood after 1.5–2 months, with very little diversification in the B cell receptor (BCR) repertoire [27,28]. Although below normal values, class switched memory B cells are observed as early as 3 months after HSCT [29], but we have limited information on the diversity of the BCR repertoire in the B cell subsets in both peripheral blood and bone marrow of HSCT-treated patients.

**Figure 3:**
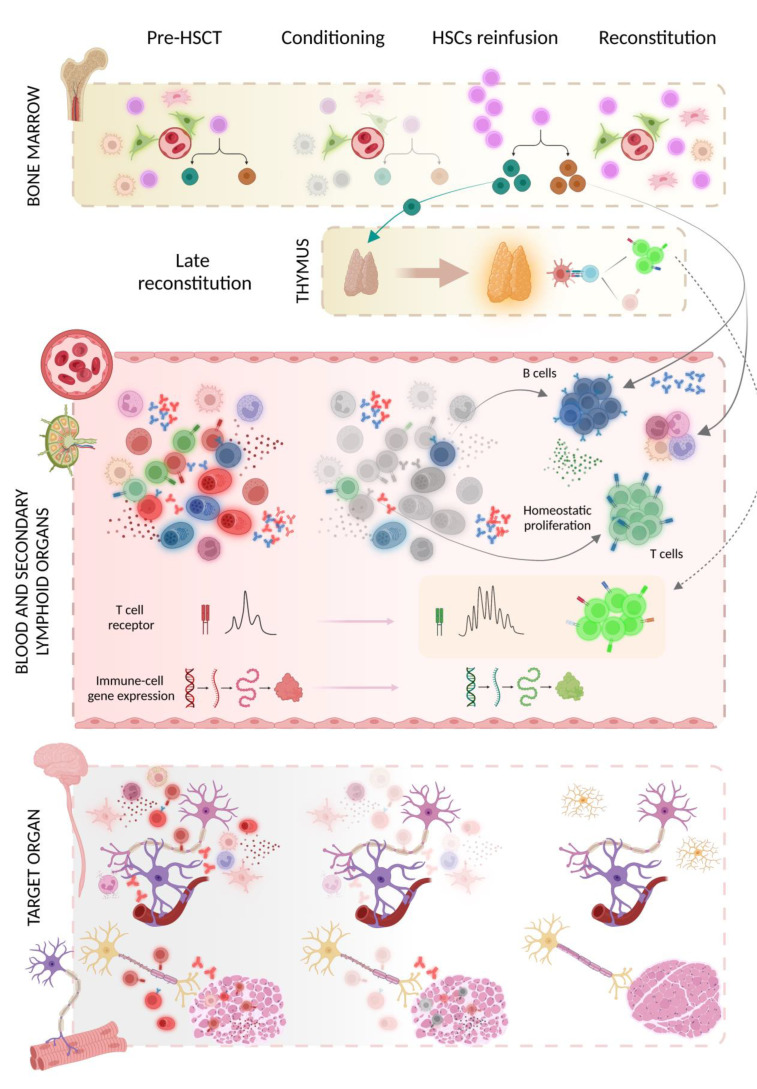
The administration of the conditioning regimen induces the elimination of circulating immune cells (including pathogenetic clones, depicted in red) and the ablation of myeloid and lymphoid progenitors in the bone marrow, to a variable extent according to its grade of intensity. Pathogenetic immune cells infiltrating the target organ may also be eliminated, and pathogenetic autoantibodies (coloured in red) disappear over time. After haematopoietic stem cells (HSCs) reinfusion, immune reconstitution occurs in two phases. During the early reconstitution (solid arrows), which takes place within 6–12 months after transplant, residual mature lymphocytes that had survived the chemotherapy or were reinfused with the graft expand by homeostatic proliferation, determining a predominance of memory cells over naïve cells and a skewing of the T cell receptor (TCR) repertoire. In addition, the differentiation of HSCs in the bone marrow promotes the repopulation of B lymphocytes and cells of the myeloid lineage. The reactivation of the thymus, seeded by lymphoid progenitors derived from bone marrow, promotes the late reconstitution (dashed arrow) that occurs one to two years after HSCT: naïve T cells are de novo generated and released to the peripheral blood after positive and negative selection in the thymus. In this phase, the reconstituted T cell pool is characterised by a new and diverse TCR repertoire compared to pre-treatment. Shifts of immune cell subsets, cytokine patterns, and expression of genes involved in immune cell function from a pro-inflammatory towards an anti-inflammatory environment contribute to the restoration of immune tolerance. Created with BioRender.com.

The phases of immunological reconstitution after HSCT and the mechanisms of action of transplant in neurological autoimmune diseases are summarised in Figure 3.

### 3.4. Safety

The safety of the procedure encompasses treatment-related side effects and death related to treatment (defined as Transplant-Related Mortality, TRM, or non-relapse mortality, NRM).

Treatment-related side effects may be further classified into early and late side effects if they occur within or beyond 100 days after transplant, respectively.

Early side effects, such as transient alopecia, gastrointestinal toxicity, and infections, are expected in the vast majority of patients, irrespective of the indication for the procedure. According to the EBMT guidelines, these are managed with the use of supportive and symptomatic treatments, antimicrobial therapy, monitoring for viral reactivation (cytomegalovirus, CMV, and Epstein-Barr virus, EBV), prophylaxis for infections (fungal, herpes virus, and pneumocystis), and pre-emptive treatments in cases of early detection of viral reactivation.

Late side effects encompass secondary autoimmunity, impairment of fertility, and secondary neoplasms.

The cumulative incidence of secondary autoimmunity, in patients treated for autoimmune indication, was 10% at year 5 of follow-up in a retrospective analysis of the EBMT registry [30]. Several risk factors were suggested, such as the primary diagnosis (higher rate for B cell-mediated autoimmune diseases, such as SLE) and the conditioning regimen used (higher risk for ATG associated with ex vivo CD34^+^ cell selection, or Alemtuzumab) [30,31]. The severity of secondary autoimmunity was mild to moderate in most cases but led to death in 2/29 patients reported in the registry study [30], strengthening the need for careful monitoring and early management. Secondary autoimmunity was observed in 3/16 cases following allo-HSCT [30].

The impairment of fertility is a frequent complication in females of childbearing potential, requiring proper counselling before transplant. Transient amenorrhea occurs in almost all cases, and it may be persistent in one-third to two-thirds of the patients. Older age at AHSCT and previous exposure to pulsed Cy were associated with a higher risk of secondary amenorrhea in MS [32]. Despite the recovery of menstruation, the ovarian reserve may be permanently affected; age, type, and cumulative dose of chemotherapeutic drugs used were identified as risk factors for secondary infertility [33]. Even if successful pregnancies have been reported following HSCT in autoimmune disorders [33,34], the pregnancy rate appears to be low, although usually not corrected for the desire for pregnancy [34].

Diagnosis of neoplasms after HSCT was reported, mostly in patients with MS, with a possible cluster for haematological malignancies. However, the causal relationship between AHSCT and cancers has yet to be determined due to the small number of incident events and the potentially relevant contribution of previous immunosuppressive treatments [35].

TRM and NRM rates in AHSCT for autoimmune diseases in general (including non-neurological disorders) and MS have dramatically reduced over time [1,36], down to an estimated 0.2% in MS [37]. The intensity of the conditioning regimen was not significantly associated to the TRM risk in an extended meta-analysis including 15 published studies [38]. Meaningful estimates of NRM risk of AHSCT in immune-mediated neurological diseases other than MS cannot be reliably provided due to small numbers, heterogeneity, and varying degrees of disability and comorbidity [1]. The same consideration applies to allo-HSCT due to the low number of treatments reported [5].

In addition to such general information, the underlying disease may confer a special susceptibility to definite adverse events, making the risk-benefit ratio of the procedure heterogeneous across different indications for transplant. Special considerations on this issue are reported in each of the following sections.

## 4. Neuromyelitis Optica Spectrum Disorder

A few retrospective and prospective non-randomised studies on HSCT in NMOSD patients have been published so far (Table 2).

The first report dates to 2010, in which a NMOSD patient with an aggressive and refractory disease course was treated with AHSCT, conditioning regimen BEAM + ATG after HSCs mobilisation with Cy, leading to clinical remission during the first 12 months post-AHSCT and improvement of the MRI abnormalities [39]. On the contrary, a case report published a year after, described a woman with NMOSD who experienced a relapse of myelitis 4 months after AHSCT for a lymphoma that developed while receiving azathioprine therapy [40]. Since then, a few case reports have been published showing favourable outcomes following AHSCT [41,42].

The efficacy of AHSCT in NMOSD was evaluated in a retrospective analysis of the EBMT registry, which included 16 NMOSD patients who underwent the procedure between 2001 and 2011 [43]. AQP4 antibody status at baseline was positive in 10/13 tested cases. Different intermediate-intensity conditioning regimens were adopted, BEAM + ATG being used in most of the cases. Progression-free survival (PFS) at years 3–5 was 48%; 81% of the patients relapsed within a median of 7 months after the procedure, with evidence of MRI activity in most of the evaluable cases. Anti-AQP4 antibodies remained positive during the follow-up in all of the 8 patients tested, indicating the persistence of the pathogenetic B cell clone despite the procedure. Over long-term follow-up, one patient died of disease progression at month 14, and four patients received a second HSCT (allogenic in 3/4 cases). After allo-HSCT, the disappearance of pathogenetic autoantibodies was reported in 2/2 tested cases, and this was associated with durable disease remission [43].

A more recent prospective study by Burt et al. showed more encouraging results for AHSCT in NMOSD patients, adopting a Cy + ATG-based conditioning regimen associated with plasmapheresis and 2 doses of rituximab [25]. PFS (defined as an increase of at least 1 EDSS point compared to baseline) was 90% at year 5. In 11 patients with NMOSD whose baseline AQP4 antibody serostatus was positive, 9 patients became seronegative up to 5 years post-AHSCT, with the complement activating and cell-killing abilities of AQP4 antibodies switched off in 6 patients after the transplant. Two patients remained AQP4-IgG-seropositive (with persistent complement activating and cell-killing ability) and relapsed within 2 years of AHSCT.

One patient, who was excluded from the analysis for a concomitant diagnosis of SLE, died following treatment due to SLE reactivation. This pilot study suggests that negativisation of anti-AQP4 antibodies could be a biomarker of treatment response. Furthermore, the choice of a “tailored” conditioning regimen based on strong B-depleting drugs could be more effective than generic immunosuppressive treatment in inducing a long-term response in NMOSD. Other perspective studies should confirm the effectiveness of rituximab-including regimens in inducing sustained remissions in NMOSD.

**Table 2:** Retrospective or prospective non-randomised studies of HSCT in NMOSD patients.

Study [Reference]	Greco, R., et al., 2015 [43]	Burt, R.K., et al., 2018 [3]	Burton, J.M., et al., 2021 [44]
**Baseline features**			
N patients; % F	16; 81% F	12; 92% F	3; 67% F
Age, y	37 (20–57)	42 (19–51)	34 (28–39)
Disease duration, y	2 (<1–17)	7 (1–19.7)	8.3 (3–13)
EDSS	6.5 (2.0–8.5)	4.3 (2–6.5)	4 (3.0–4.5)
ARR before AHSCT	N.R.	4.3 (2–10)	3.4 (1.3–5) in the y pre-AHSCT
Anti-AQP4 ab positive	10/13 tested (62%)	92%	67%
**AHSCT protocol**			
Mobilisation of HSCs	Cy (2–4 g/m^2^) + G-CSF (+ RTX 375 mg/m^2^ in 2 cases)	Cy 2 g/m^2^ + G-CSF	Cy 2 g/m^2^ + RTX 375 mg/m^2^ + G-CSF
Conditioning	BEAM + ATG (n = 9); thiotepa-Cy (n = 3); Cy 200 mg/Kg + ATG (n = 4)	Cy 200 mg/Kg + ATG + RTX 500 mgx2 + plasmapheresis ^1^	Cy 200 mg/Kg + ATG + RTX 375 mg/m^2^
**Outcomes**			
Progression-free survival	48% at y 3–5	90% at y 5	67% at last follow-up
EDSS	improved in 56% ^2^	3.0 (0–6.5) at y 5	improved in 67% ^2^
Relapse-free survival	31% at y 3; 10% at y 5	80% at y 5	33% at last follow-up
IS-medications free	19%	83%	33%
Anti-AQP4 ab positive	8/8 tested (100%)	17%	67%
Severe adverse events and death	one death due to disease progression at month 14; one grade 4 neutropenia	no grade 4 toxicities	1 death due to disease progression at y 3.5
Secondary autoimmunity	1 thyroiditis	1 MG; 1 hyperthyroidism	N.R.
Follow-up duration, y	4 (1.7–10.7)	4.7 (2–5)	7.5 (3.5–10)

^1^ performed before hospital admission; ^2^ comparisons between the last follow-up and baseline. Continuous variables are reported as median/mean (range), where applicable. Abbreviations: AQP4 ab, anti-aquaporin 4 antibodies; ARR, annualised relapse rate; ATG, anti-thymocyte globulin; Cy, cyclophosphamide; G-CSF, granulocyte-colony stimulating factor; IS, immunosuppressive; MG, myasthenia gravis; N.R., not reported; RTX, rituximab; y, years.

A prospective non-randomised single-arm trial showed heterogeneous outcomes in the only 3 patients enrolled due to recruitment failure [44]. Patients were mobilised with Cy plus rituximab 375 mg/m^2^ and conditioned with Cy + ATG and a second dose of rituximab 375 mg/m^2^. Suppression of relapses with disability improvement after 10 years was reported in 1 case that was anti-AQP4 antibody negative at baseline. The remaining two patients showed disease reactivation within 2 years from transplant, requiring initiation of immunosuppressive treatment and leading to death in one case due to relapse-induced hypothalamic failure. Both of these patients were anti-AQP4 antibody positive at baseline and remained positive over follow-up. The overall safety of the procedure was acceptable, with only minor treatment-related adverse events. A more comprehensive summary of the state-of-the-art in this field, including reports on allo-HSCT, has recently been provided, along with a systematic review and meta-analysis [45,46].

## 5. Stiff-Person Syndrome

SPS is a rare autoimmune neurological disorder characterised by fluctuating rigidity and stiffness of the axial and proximal lower limb muscles, with superimposed painful spasms and continuous motor unit activity on electromyography [47]. SPS may have heterogeneous manifestations, including progressive encephalomyelitis with rigidity and myoclonus (PERM) [48]. Antibodies directed against glutamic acid decarboxylase (GAD65) are detected in 70–80% of the cases, but different autoantibodies are also described. Multiple mechanisms of immune-mediated damage, driven by multiple immune specificities, can exist within the same patient [49]. Conventional treatments include symptomatic drugs and immunomodulant agents, mostly corticosteroids, plasma exchange, intravenous immunoglobulins (IVIG), and rituximab, even if the latter failed to show efficacy superior to placebo in a randomised clinical trial [50,51,52]. Despite the combination of multiple symptomatic and immunological therapies, more than half of the patients require long-term mobility aids [53].

The first report on AHSCT in anti-GAD65+ SPS was published in 2014, describing the outcome of two female patients treated in 2009 and 2011 with the high-intensity protocol Busulfan-Cy + ATG and CD34^+^ selection [54]. The patients were affected by severe disease, leading to a loss of working ability and social withdrawal, despite receiving symptomatic treatment and immunomodulation. Over a post-AHSCT follow-up of 56 months and 32 months, both patients achieved clinical remission with sustained and marked improvement in symptoms and a return to premorbid functioning, without any immunomodulant treatments and without discontinuation or tapering of symptomatic medications. Anti-GAD antibody titre was markedly reduced in one case, but the persistence of circulating antibodies did not correlate with clinical disease activity.

Since then, two case series and a prospective single-arm trial, all adopting intermediate-intensity conditioning AHSCT, have been published (Table 3).

Between May 2014 and July 2016, nine patients with severe SPS confirmed by EMG and elevated anti-GAD autoantibodies were treated with BEAM + ATG AHSCT, after HSCs mobilisation with rituximab + G-CFS [55]. All the patients required assistance for ambulation and were unable to work. By 1 to 2 years post-AHSCT, all the cases had achieved significant and sustained improvement in the median distribution of stiffness index and functional status, with tapering or discontinuation of symptomatic medications in most of the cases. This was associated with an improvement or normalisation of EMG findings in all the patients and a reduction in anti-GAD titres. No fatalities occurred, and no severe or unexpected adverse events were reported.

More recently, four patients (three classical SPS and one PERM) treated with Cy + ATG protocol between 2015 and 2019 were described in the literature [56]. All the patients were significantly disabled, had failed conventional immunosuppressive therapy, and showed typical SPS features at electrophysiological tests. Anti-GAD antibody positivity was detected in all the cases, with high titres (>2000 U/mL) in patients with a classical phenotype and a lower titre (372 U/mL) associated with anti-glycine and anti-gliadin antibody positivity in the PERM. Over a follow-up of 12–36 months, all the patients showed clinical improvement and were free from immunosuppressive therapies. Ambulation improved remarkably in all the cases, and two wheelchair-dependent patients regained the ability to ambulate independently. The normalisation of the electrophysiological abnormalities and negativisation for anti-GAD antibodies were observed in two cases. Adverse events were aligned with the routine toxicity of the procedure, with in-hospital stays ranging from 16 to 26 days.

**Table 3:** Prospective non-randomised studies and case series of HSCT in SPS patients.

Study [Reference]	Sanders, S., et al., 2014 [54]	Kass-Iliyya, L., et al., 2021 [56]	Georges, G.E., et al., 2018 [55]	Burt, R.K., et al., 2021 [57]
**Baseline features**				
N patients; % F	2; 100% F	4 (3 SPS, 1 PERM); 75% F	9; 44% F	23; 91% F
Age, y	A: 53; B: 33	43 (36–52)	42 (25–50)	48 (28–60)
Disease duration, y	A: 5; B: 5	6.5 (4–9)	5.3 (1–14.7)	7 (2–20)
Need for assistance in gait	50% (case A)	75% wheelchair dependent; 25% restricted walking	100%	78%
Anti-GAD ab positive	100% in serum	100% in serum	100% in serum	100% in serum; 14/20 (70%) in CSF
EMG abnormalities	normal in A; continuous motor unit activity in B	100% (continuous motor unit activity in 3/4, blink reflex hyperexcitability in 4/4)	100%	70% (continuous paraspinal muscle activity)
**AHSCT protocol**				
Mobilisation of HSCs	Cy 2.5 g/m^2^ + G-CSF	Cy 2 g/m^2^ + G-CSF	rituximab + G-CSF	Cy 2 g/m^2^ + G-CSF
Conditioning	Busulfan–Cy + ATG + CD34^+^ selection	Cy 200 mg/kg + ATG	BEAM + ATG	Cy 200 mg/kg + ATG + RTX 500 mg ×2
**Outcomes**				
Clinical outcome	SPS symptoms	improvement in walking	improvement in distribution of stiffness index and functional status	discontinuation of immune medication and ≥50% decrease in antispasmodic medications
Rate or response	resolution in case A; improvement in case B	100%	100%	48% responders; 26% partial responders
Anti-GAD ab	titre reduced in case A; N.A. in B	negativisation in 50%	titres decreased	titres decreased in 17%
EMG	N.R.	abnormal in 50%	improved or normalised in 100%	N.R.
Tapering/discontinuation of anti-spasmodic drugs	discontinuation in A; tapering in B	75% (1 discontinuation; 2 tapering)	majority of the cases	61% (3 discontinuation; 11 tapering)
Immunotherapy-free	100%	100%	N.R.	43%
Severe adverse events and death	none	none	none	1 death due to disease progression at y 1; grade 4 tox in 3 cases
Secondary autoimmunity	none	none	N.R.	1 hypothyroidism at y 2
Follow-up duration, y	A: 5; B: 3	1.7 (1–3)	1 to 2	3.6 (1.5–4.5)

Continuous variables are reported as median/mean (range), where applicable. Abbreviations: anti-GAD ab, anti-glutamic acid decarboxylase antibodies; ATG, anti-thymocyte globulin; Cy, cyclophosphamide; G-CSF, granulocyte-colony stimulating factor; N.R., not reported; RTX, rituximab; y, years.

Less encouraging results were provided by a prospective single-arm trial started in 2014, aimed at evaluating the safety of the procedure as the primary outcome [57]. Anti-GAD positivity in the serum and/or CSF and EMG findings consistent with SPS were required for inclusion. The conditioning protocol was Cy + ATG and rituximab 500 mg on days −6 and +1. Twenty-three patients were included. No TRM occurred, and no unexpected transplant-related toxicities were observed, but the enrolment was prematurely terminated due to evidence of short-term response or lack of response at all in a subgroup of cases, in the absence of factors predictive of positive outcomes. Treatment response was defined as both discontinuation of immune medication and at least a 50% decrease in antispasmodic medications.

Patients who initially met the responder criteria but then relapsed and needed further immune medications and/or restarted or increased antispasmodic drugs were defined as partial responders. According to these definitions, 11 cases (48%) were considered responders, and six cases (26%) were considered partial responders or non-responders. Need for assistance in gait was reported in 18/23 cases at baseline and in 12/23 at follow-up, with an improvement compared to the pre-treatment status in 11/23 cases. One patient died one year following the transplant due to disease progression. After transplant, anti-GAD antibody titres were reduced or negative in 4 cases, but their persistence did not correlate with clinical outcomes.

A recent editorial on the latter study highlighted some limitations, such as the lack of a dependency test for IVIG to confirm an active disease and of assessment of spasms with dedicated clinical scales [58]. Moreover, the authors performed retrospective data mining on clinical parameters that should not be considered reliable predictive factors of treatment response. Despite these limitations, the study suggested that AHSCT may be well-tolerated and safe in SPS patients and strengthened the opportunity for further exploring it in SPS with controlled trials designed with more objective assessments.

## 6. Chronic Inflammatory Demyelinating Polyneuropathy

CIDP is a slowly progressive autoimmune demyelinating disease of the peripheral nervous system that evolves over two months with a relapsing-remitting course or a step-wise progression [59].

The first AHSCT for CIDP was performed in 2002 in a 48 years-old man, affected by severe disease requiring continuative immunosuppressive treatments (corticosteroids, IVIG, azathioprine and methotrexate) at high doses that were no longer tolerated due to severe side effects [60]. The manipulated graft (ex-vivo CD34^+^ selection) was reinfused after conditioning with BEAM. After AHSCT, an improvement in the neurophysiological testing was observed, with disease remission occurring without IVIG administration (low-dose prednisone was maintained, probably due to adrenal insufficiency). However, after 5 years, the patient experienced a relapse of the disease associated with worsening of the electrophysiological findings that required further IVIG courses, even if at a lower dosage than previously needed [61].

Since then, a few case series/case reports and one prospective study exploring the role of transplant in CIDP have been published, with about 140 patients undergoing treatment (Table 4).

Thirty-four patients were reported across four studies mainly adopting Cy + ATG as a conditioning regimen [62,63,64,65]. Inclusion criteria were slightly different, and treatment failure was not required in all the studies [62].

**Table 4:** Prospective non-randomised studies and case series of HSCT in CIDP patients.

Study [Reference]	Mahdi-Rogers, M. et al., 2009 [62]	Ajroud-Driss, S. et al. 2011, ^1^ [64]	Press, R., et al., 2013 [63]	Burt, R.K., et al., 2020 [66]	Masson-Roy, J., et al., 2021 [65]
**Baseline**					
N patients; % F	3; 66% F	15; 47% F	11; 9% F	66 treated, 60 analysed; 38% F	5; 20% F
Age, y	58 (29–72)	39 (24–64)	55 (23–68)	43 (20–63)	48 (28–60)
Disease duration, y	13 (7–21)	N.R.	2.5 (<1–19)	4.7 (<1–29)	7 (2–20)
**AHSCT protocol**					
Mobilisation of HSCs	Cy 4 g/m^2^ + G-CSF	N.R.	Cy 2–4 g/m^2^ + G-CSF (n = 9); RTX 375 mg/m^2^ + G-CSF (n = 2)	Cy 2 g/m^2^ + G-CSF	Cy 2.5 g/m^2^ + G-CSF
Conditioning	Cy 200 mg/Kg + ATG	Cy + ATG and CD34^+^ cells selection	Cy 35–50 mg/kg alone (n = 1) or + ATG (n = 6); melphalan (n = 1); BEAM + ATG (n = 3)	Cy 200 mg/Kg + ATG + RTX 500 mg on days −6 and +1	busulfan + Cy + ATG (n = 3); BEAM (n = 2). + CD34^+^ cells selection (n = 4)
**Outcomes**					
Clinical measures	Clinical improvement 1/3 (33%), worsening 2/3 (67%). Median MRC sum score from 50.3 to 44.3	Remission in 9/14 (64%) with significant improvement in strength; worsening in 1/14 (7%); 1 lost at follow-up	Significant improvement in the median INCAT (1) and Rankin score (1) compared with baseline (6 and 4, respectively)	Improvement in unassisted ambulation (32% → 83% at y 4–5). Immune drugs-free remission: 78% at y 4 and 83% at y 5	Clinical improvement in 4/5 (80%); stabilisation in 1/5 (20%)
Electrophysiological parameters	Improvement in one case; N.R. in 2	improvement in distal latency, and/or NCV and/or CMAP in 8/11 (73%)	Improvement in the median CMAP (1.84 mV compared to 0.88 mV)	Improvement in NCV (from 27 to 38) and CMAP (from 3.5 to 4.1) for all nerves	Trend towards improvement in most nerve conduction studies
Cases with relapse (time of relapse)	1 (month 18)	1	3 (months 23, 14 and 14)	11 (19%)	0
Immune-medications	weekly IVIG and oral prednisolone (n = 1)	discontinuation in 9/14 (64%), tapering in 4 (36%)	AHSCT (n = 1); tocilizumab (n = 1); oral steroids (n = 2)	restart of IVIG, PLEX, orRTX in 11/60 (18%)	1 (20%) hydrocortisone due to adrenal insufficiency
Severe adverse events and death	1 severe pneumonia requiring intensive care	no major side effects	Klebsiella, Pseudomonas and α-Streptococci sepsis (1); pancreatitis (1)	3 grade 4 early toxicities; two deaths considered not transplant-related.	no grade 4 toxicities
Follow-up, months	19 (6–25)	6 (3–62)	28 (6–127)	54 (24–60)	41 (11–119)

^1^ conference abstract. Continuous variables are reported as median/mean (range), where applicable. Abbreviations: ATG, anti-thymocyte globulin; CMAP, Compound Muscle Action Potential; Cy, cyclophosphamide; G-CSF, granulocyte-colony stimulating factor; INCAT, Inflammatory Neuropathy Cause and Treatment; IVIG, intravenous immunoglobulins; MRC, Medical Research Council; NCV, nerve conduction velocity; N.R., not reported; PLEX, plasma-exchange; RTX, rituximab; y, years.

After AHSCT, clinical improvement was reported over midterm follow-up in most cases. Relapses were reported in 5 patients [62,63,64], and this was usually followed by a better response to immunomodulatory treatments than before the transplant.

No TRM was reported; severe events occurred in 5 patients (intensive care treatment; 2 cases of pneumonia; pancreatitis, and gastrointestinal bleeding).

An interim report in 2014 described an improvement in the Modified Rankin scale and Short Form Health Survey 36 (SF-36) questionnaire in most of the 32 patients treated, with a drug-free remission in 67% of the cases after 2 years of follow-up [67].

The largest case series published to date included 60 patients (37 males) with a median age of 43 years, and a history of at least one previous disease-specific treatment in 60 cases [66]. At baseline, the median Inflammatory Neuropathy Cause and Treatment (INCAT) Score was 6 (1–9), Rankin Score was 3.0 (2–4), and the Barthel Score 83/85 (30–100). The conditioning protocol was Cy + ATG; rituximab 500 mg was added at day −6 and +1. Over a mean follow-up of 4.5 years, a significant improvement was observed both in clinical and electrophysiological measures. Post-transplant immune medication-free remission was 80% and 78% at years 1 and 4, respectively, and a remarkable increase in cases able to ambulate without assistance (from 32% pre-HSCT to more than 80% at follow-up) was observed. There was no TRM, and overall survival was 97%. As for side effects, three patients experienced grade 4 toxicities.

Interestingly, a study from the same authors reported that AHSCT was cost-effective compared with chronic IVIG, with estimated projected health care savings of up to $438,054 per patient over a 5-year period [68].

Overall, these studies suggest that AHSCT may be safe and effective for treatment-refractory CIDP.

## 7. Myasthenia Gravis

MG is an autoimmune disease of the neuromuscular junction characterised by the presence of skeletal muscle weakness caused by antibodies directed against AChR, muscle-specific kinase (MUSK), or low-density lipoprotein receptor-related protein 4 (LRP4) [69]. The therapy is based on symptomatic drugs, with acetylcholinesterase inhibitors, and an immunosuppressant treatment, with a broad spectrum of agents ranging from corticosteroids and plasma exchange to new biological agents [70]. HSCT has rarely been used for MG, and it has been reserved for refractory cases.

Twelve cases of refractory MG treated with HSCT are reported in the literature (Table 5) [69,70,71,72,73,74,75,76]. Only one patient received allo-HSCT from an HLA-matched sibling [74], while all the others had AHSCT. MG antibody status was positive for anti-AchR for 8 patients, and anti-MuSK for one patient, while the remaining two patients were seronegative for AchR antibodies (MuSK antibodies were not tested).

Stem cell mobilisation was obtained with Cy and G-CSF in nine patients and with rituximab and G-CSF in another patient [73]. Then, immune ablation was obtained with high-intensity conditioning regimens in seven patients, an intermediate-intensity conditioning regimen in two patients [72,75] and a reduced-toxicity conditioning regimen in another patient [74].

The main side effects reported were mucositis, neutropenic fever, transient viral reactivations, bacteraemia, urinary tract infections, and a secondary autoimmune disease in one patient (acquired amegakaryocytic thrombocytopenia). MG symptoms with worsening of muscle weakness and respiration, which required invasive or non-invasive ventilation and immunosuppression, were reported in two patients [73].

**Table 5:** Case report and case series of HSCT in MG patients.

Study [Reference]	Bryant, A., et al., 2016 [71]	Sossa Melo, C.L. et al., 2019 [72]	Inan, B., et al., 2022 [75]	Håkansson, I. et al., 2016 [73]	Mitsumune, S. et al., 2018 [76]	Strober, J. et al., 2009 [74]
N cases (gender)	7 (6F, 1M)	1 (M)	1 (F)	1 (F)	1 (M)	1 (M)
Age, y	24–55	56	26	64	54	17
HSCT	autologous	autologous	autologous	autologous	autologous	allogenic
Mobilisation of HSCs	Cy + G-CSF	Cy + G-CSF	Cy + G-CSF	RTX + G-CSF	N.R.	N.R.
Conditioning	Cy + TBI + ATG (n = 2); busulfan-Cy + ATG (n = 4); etoposide, melphalan + TBI (n = 1)	Cy + ATG	Cy + ATG	BEAM + ATG	N.R.	Busulfan + fludarabine + alemtuzumab
Outcome	remission	remission	partial response	partial response	remission	partial response
Follow-up, m	N.R.	65	30	24	N.R.	40

Continuous variables are reported as median/mean (range), where applicable. Abbreviations: ATG, anti-thymocyte globulin; Cy, cyclophosphamide; F, female; G-CSF, granulocyte-colony stimulating factor; m, months; M, male; N.R., not reported; RTX, rituximab; TBI, total body irradiation; y, years.

Complete stable remission of MG, with therapy withdrawal, was obtained in nine patients, with a median follow-up of 40 months. Two patients continued to have only ocular symptoms, with AChR antibodies still detectable, although reduced [73,74]. Another patient with MG and familial mediterranean fever obtained a reduction in the frequency and severity of myasthenic exacerbations but had to continue immunosuppressive therapy [75].

HSCT proved to be a valid treatment option in severe cases of refractory MG, but systemic toxicity, infection risk, and infertility have to be considered. A clinical trial investigating the use of HSCT in refractory MG was started in 2002 (NCT00424489), but it was prematurely terminated because of failure of enrollment, and three deaths out of the first nine treated cases were reported [77].

## 8. Inflammatory Myopathies

Few cases of inflammatory myopathies treated with high-dose chemotherapy followed by HSCs reinfusion are reported, including patients affected by polymyositis, dermatomyositis, and sporadic late-onset nemaline myopathy (SLONM).

Polymyositis and dermatomyositis are inflammatory myopathies that present with symmetrical, proximal muscle weakness, associated with skin involvement in the latter, and are treated with steroids and immunosuppressive drugs with a variable response [78]. One case of Jo-1-associated polymyositis successfully treated with busulphan and Cy was reported [79]. Five cases of refractory juvenile dermatomyositis who underwent AHSCT were described [80,81]. The conditioning regimen used was fludarabine, Cy, and ATG [80] or Cy plus ATG [81]. A dramatic improvement and sustained drug-free remission of the disease were reported in all the cases at variable follow-up (from 13 to 144 months).

SLONM is a rare adult-onset disorder, characterised by predominant limb-girdle and axial weakness with atrophy, with a probable immune-mediated pathogenesis [82]. Treatment with high dose melphalan followed by autologous HSCs reinfusion was reported in nine patients with SLONM and an associated monoclonal gammopathy of unknown significance (MGUS) [83,84]. A moderate-to-good clinical response was observed in most cases, although re-treatment was required in two patients. Two non-transplant-related deaths (Salmonella typhi septicaemia at month 5 and disease progression at year 5 of follow-up) were reported [83,84].

The use of allo-HSCT was also reported as a potentially promising therapeutic approach for the treatment of genetic disorders such as mitochondrial neurogastrointestinal encephalomyopathy syndrome (MNGIE) [85,86,87] and myositis in Griscelli syndrome type 2 [88].

## 9. Rare Neurological Disorders and Systemic Autoimmune Diseases with Neurological Involvement

Systemic rheumatologic diseases, such as SLE, Behcet disease, and systemic vasculitis, are a broad spectrum of autoimmune disorders that often involve multiple organ systems, including the CNS and peripheral nervous system, and may cause a variety of neurological manifestations. They also represent a challenge in management and treatment, requiring multidisciplinary expertise in diagnosis and therapy to minimise the risk of permanent neurologic deficits [89].

HSCT has been widely used in rheumatologic disorders; here we reported only specific cases where a refractory neurologic syndrome was the indication to perform HSCT (Table 6).

Six cases of Behcet disease with refractory neurologic involvement underwent AHSCT [90,91,92,93]. HSCs mobilisation was obtained with Cy and G-CSF in five cases and with Cy alone in the remaining case. The conditioning was performed using Cy and ATG in two cases, BEAM in three cases, and melphalan alone in another case. The main adverse reactions were neutropenic fever and herpes zoster in one patient. Outcomes were good, with immunotherapy withdrawal and remission of neurologic manifestations in three cases, while another patient remained stable in the disability already achieved but didn’t experience new relapses after the immunosuppressive treatment withdrawal. In one case, the disease progressed after AHSCT. Another patient experienced a severe relapse within three months from transplantation, which required an allo-HSCT that was complicated by GVHD and followed by another relapse after 2 years.

The literature also reports a case of Sjogren syndrome (SS) and one of Wegener granulomatosis (WG), both with neurologic involvement, who underwent AHSCT [93]. Further details are reported in Table 6. Cy and G-CSF were used for HSCs mobilisation and Cy and ATG were used for conditioning. As side effects, neutropenic fever was reported in both patients; the patient with SS had a flare of optic neuritis during HSCs mobilisation, and the patient with WG reported CMV and EBV reactivation, urinary tract infection, and oral thrush. Both patients achieved remission, and they could discontinue immunosuppressive therapy.

AHSCT has been used and studied in patients with SLE by Burt and colleagues. In the first clinical trial to evaluate AHSCT in refractory SLE, in a cohort of 50 patients, 32 had neurologic involvement, and in 18/32 cases, the neurologic manifestation was the primary indication for HSCT. HSCs mobilisation was achieved using Cy and G-CSF, and the conditioning regimen consisted of Cy and ATG. At year 5, overall survival and the probability of disease-free survival were 84% and 50%, respectively. HSCT also showed a disease-ameliorating effect [94].

**Table 6:** Case reports on HSCT in rare neurological diseases and autoimmune systemic disorders with neurological involvement.

Study [Reference]	N cases (Gender)	Age	Mobilisation of HSCs	Conditioning	Outcome	Follow-Up
**Behcet Disease**						
Statkute, L. et al. [93]	2 (F)	25, 36	Cy + G-CSF	Cy + ATG	(1) remission, (2) no response	28 m
De Cata, A. et al., 2007 [91]	2 (M)	22, 23	Cy + G-CSF	BEAM	partial response	48 m
Marmont, A.M. et al., 2006 [92]	1 (F)	34	Cy	BEAM	severe relapse < 3 m → allogenic HSCT with relapse at y 2	
Daikeler, T. et al., 2007 [90]	1 (M)	49	Cy + G-CSF	Melphalan	partial response	
**Sjogren syndrome**						
Statkute, L. et al., 2008 [93]	1 (F)	42	Cy + G-CSF	Cy + ATG	remission	28 m
**Wegener granulomatosis**						
Statkute, L. et al., 2008 [93]	1 (F)	27	Cy + G-CSF	Cy + ATG	remission	28 m
**Systemic lupus erythematosus**						
Burt, R.K. et al., 2006 [94]	18		Cy + G-CSF	Cy + ATG	response	5 y
Lehnhardt, F.G. et al., 2006 [95]	1 (F)	19	Cy + G-CSF	Cy + ATG	remission	18 m
Trysberg, E. et al., 2000 [96]	1 (F)	19	Cy + G-CSF	Cy + TBI	remission	18 m
Goklemez, S. et al., 2022 [97]	3 (1M, 2F)	33, 20, 15	Cy + G-CSF + RTX	Cy + fludarabine + RTX	(1,2) remission, (3) relapse at 18 m	162 m
Lisukov, I.A. et al., 2004 [98]	2 (F)	21, 25	(1) collected from BM, (2) mobilised with Cy + G-CSF	(1) etoposide + melphalan, (2) Cy	remission	45 m, 6 m
**Autoimmune encephalitis**						
Froehlich, M. et al., 2020 [99]	1 (F)	35	Cy	Cy + ATG	remission	18 m

Abbreviations: ATG, anti-thymocyte globulin; Cy, cyclophosphamide; F, female; G-CSF, granulocyte-colony stimulating factor; m, months; M, male; RTX, rituximab; y, years.

Seven specific cases of neuro-SLE, for whom neurologic involvement was the indication for AHSCT, are reported in the literature [95,96,97,98]. HSCs mobilisation was performed in three patients using Cy and G-CSF, while in the other three cases, rituximab was added to this regimen. In one patient, HSCs were collected from bone marrow. Different conditioning regimens were used (Table 6). Reported adverse events included common transplant toxicities, haemolytic anaemia, appendicitis, and a transient worsening of transverse myelitis. Six patients showed a complete response with neurological improvement and absence of SLE activity at 18+ months, while one patient had only a partial response with a CNS relapse at 18 months. In the responders’ group, one patient had a skin and renal relapse at 191 months, and another one died at 162 months from unknown causes.

## 10. Conclusions

AHSCT is currently recognised by the EMBT as a “standard of care” in highly active relapsing-remitting MS that fails disease-modifying treatments [100].

Growing evidence supporting the role of HSCT in the treatment of carefully selected patients with other autoimmune neurological diseases refractory to conventional therapies is provided by case report/series and a few phase II studies. The results were overall encouraging, although the risk-benefit ratio was deemed uncertain in some diseases, such as MG, and a possible publication bias should be considered.

The selection of conditioning regimens “tailored” to the pathogenetic mechanism of the disease is probably relevant in determining favourable outcomes, as previously suggested [101].

According to the recommendations reported in the EBMT guidelines published in 2019 and in the eight EBMT reports on indications for transplant, AHSCT is considered a “clinical option” supported by grade II evidence for CIDP, NMO, MG, and SPS, as well as for systemic autoimmune diseases with neurologic manifestations [1,100]. This means that “HSCT is a valuable option for individual patients after careful discussions of risks and benefits with the patient but the value of HSCT needs further evaluation in such groups of patients”, as the results of small patient cohorts show efficacy and acceptable toxicity of the procedure, but confirmatory randomised studies are missing, often as a result of the low number of patients treated [1]. On the other hand, allo-HSCT was considered “generally not recommended” for neurological autoimmune diseases, with the only exception of NMO, where grade III evidence suggests a “developmental” indication, i.e., these transplants should be performed within the framework of a clinical protocol approved by local research ethics committees and complying with current international standards (normally undertaken by transplant units with acknowledged expertise in the management of that particular disease or that type of HSCT), as the experience is limited and additional research is needed to define the role of HSCT. Updated EBMT guidelines on the use of HSCT during the COVID-19 pandemic confirm its role as a “clinical option” (grade II evidence) for treatment-resistant NMOSD, MG, CIDP and SPS spectrum disorders, as well as for rare neurological immune-mediated disorders [102].

Further evidence on this topic is awaited from prospective and ongoing studies, summarised in Table 7.

## Figures and Tables

**Table 1:** Pre-clinical bone marrow transplantation studies.

Study [Reference]	Animal Model	HSCT Protocol	Evaluation Time after BMT	Clinical Evaluation and Outcomes	Histopathological/Laboratory Assessment
Ikehara, S. et al., 1985 [8]	Mice; strains with spontaneous SLE-like disease	Conditioning: TBI (850–950 rads from a cobalt-60 source) Reconstitution: IV injection of 2 × 10^7^ allogenic BM cells from young healthy mice BMT either (1) before or (2) after AD onset	3 and 5 months	(1) Mice in pre-AD state (BMT before disease onset): no development of thymic abnormalities and AD up to month 5; (2) Mice with evidence of AD and lymphadenopathy at BMT: survival beyond month 3 after BMT.	(1) no development of thymic abnormalities (histological examination); (2) disappearance of lymphadenopathy and marked amelioration of lymphoid cell infiltrations into the kidney and liver; marked reduction of glomerular deposits of IgG, IgA, C3, and gp70 (immunofluorescence); reduced levels of CICs and anti-dsDNA ab.
van Bekkum, D. W. et al., 1989 [9]	Rats; inbred strains Buffalo and Wag/Rij, with induced AA ^a^	Conditioning: TBI (850 cGy) Reconstitution: IV injection of 5 × 10^7^ BM cells from syngeneic (Buffalo) or allogeneic (Wag/Rij) sex-matched donors BM cells collection: suspension by flushing of femoral bones cavities with Hanks’ salt solution	Weekly observation (arthritic score) up to: −14 w after syngeneic BMT −9 w after allogenic BMT.	Clear-cut complete regression of arthritis (expressed as a gradual decrease of paw thickness) in BMT-treated groups compared to controls.	Absence of inflammatory reaction in treated rats at 4 weeks after BMT versus classical severe inflammation and destruction process in control rats (histological examination).
Karussis, D. M. et al., 1992 [12]	SJL/J mice with induced EAE ^b^	Conditioning regimens (three groups): (1) TBI (900 cGy) (2) TBI (1100 cGy) (3) Cy (300 mg/kg) Reconstitution: syngeneic BM cells	3 months	(1) delayed onset and reduction in incidence and severity of EAE; (2) reduced incidence of EAE (developed in 1/7 treated mice); (3) no development of EAE, and resistance to rechallenge with the same encephalitogenic inoculum.	Lymphocytes obtained from treated mice did not proliferate in vitro in response to myelin basic protein or tuberculin-purified protein derivative, contrary to controls.
van Gelder, M. et al., 1993 [13]	Buffalo rats with induced EAE ^c^	Conditioning: TBI (900 cGy) Reconstitution: syngeneic BM cells from healthy donors.	-	In mice with already developed severe paresis before BMT, greatly accelerated recovery of paresis compared with untreated controls.	N.R.
Pestronk, A. et al., 1983 [14]	Female Lewis rats with induced EAM ^d^	Conditioning: TBI (600 rads from a dual-source cesium 137 irradiator) + Cy (200 mg/kg) Reconstitution: IV reinfusion of 6 × 10^7^ autologous BM cells BM cells collection: autologous femur, tibia, spleen, and lymph nodes, washed twice and suspended in RPMI.	8 weeks	N.R.	Prompt and sustained fall in the levels of serum ab against both foreign *(Torpedo),* and self (rat) AChR: −titre of ab against Torpedo AChR reduced to 2% of pre-treatment levels at 8 weeks; −titre of auto-ab against rat AChR fell to undetectable levels within 2–3 weeks and did not rise subsequently.

^a^ AA obtained with the intradermal injection of a suspension of M. tuberculosis in incomplete Freund’s adjuvant. ^b^ EAE induced by immunisation with spinal cord homogenate in adjuvant. ^c^ EAE induced by immunisation with syngeneic spinal cord homogenate in complete adjuvant. ^d^ EAMG obtained by immunisation with a first injection of 50 μg of AChR (from the electric organ of *Torpedo californicus)*, emulsified in 50 μg adjuvant and rechallenge injections. In this model, rats did not develop weakness but expressed AChR ab. Abbreviations: AA, adjuvant arthritis; ab, antibodies; AChR, anti-acetylcholine receptor; AD, autoimmune disease; BM, bone marrow; BMT, bone marrow transplantation; cGy, Centigray; Cy, cyclophosphamide; EAE, experimental autoimmune encephalomyelitis; EAMG, experimental autoimmune myasthenia gravis; CICs, circulating immune complexes; Ig, immunoglobulin; N.R., not reported; SLE, systemic lupus erythematosus; TBI, total-body irradiation; w, weeks.

**Table 7:** Prospective studies investigating the use of HSCT in autoimmune neurological diseases (studies focusing on MS are excluded).

Study (NCT); Phase [Reference]	Diseases Included	Conditioning Regimen	Estimated Enrolment (n Participants)	Start Date	Estimated Completion Date	Status
Autologous Peripheral Blood Stem Cell Transplant for Neurologic Autoimmune Diseases (NCT00716066); phase II [103]	Primary CNS vasculitis Rasmussen’s encephalitis CIDP; Autoimmune peripheral neuropathy Autoimmune cerebellar degeneration Gait Ataxia with Late age Onset Polyneuropathy SPS MG; Lambert-Eaton myasthenic syndrome HTLV-1-associated myelopathy/tropical spastic paraparesis Opsoclonus/myoclonus MS; Neuromyelitis optica Other central or peripheral nervous system autoimmune diseases as approved by study neurologists and faculty	BEAM + ATG followed by autologous or syngeneic stem cell transplantation	80	June 2008	June 2023	Recruiting
Stem Cell Transplantation in Idiopathic Inflammatory Myopathy Diseases (NCT00278564); phase I [104]	Polymyositis, Dermatomyositis, Juvenile polymyositis/dermatomyositis, Myositis associated with other collagen diseases	Cy + ATG + rituximab	7	September 2005	July 2016	Terminated (high relapse rate)
Hematopoietic Stem Cell Therapy for Patients With Refractory Myasthenia Gravis (NCT00424489); phase I [77]	MG	Cy + ATG	9	February 2002	June 2016	Terminated (No plan to continue study)

Abbreviations: ATG, anti-thymocyte globulin; CIDP, Chronic Inflammatory Demyelinating Polyneuropathy; Cy, cyclophosphamide; HTLV-1, Human T cell lymphotropic virus; MG, myasthenia gravis; MS; multiple sclerosis; SPS, Stiff-person syndrome.

## References

[1] Sharrack B., Saccardi R., Alexander T., Badoglio M., Burman J., Farge D., Greco R., Jessop H., Kazmi M., Kirgizov K. (2019). Autologous haematopoietic stem cell transplantation and other cellular therapy in multiple sclerosis and immune-mediated neurological diseases: Updated guidelines and recommendations from the ebmt autoimmune diseases working party (adwp) and the joint accreditation committee of ebmt and isct (jacie). Bone Marrow Transplant..

[2] Saccardi R. (2022). Personal communication.

[3] Burt R.K., Balabanov R., Han X., Morgan A., Clendenan A., Calvario M.A., Henry J., Quigley K., Gastala J., Jovanovic B. (2018). Autologous non-myeloablative hematopoietic stem cell transplantation in patients with neuromyelitis optica spectrum disorder (nmosd): An open-label pilot study. Neurology.

[4] Cohen J.A., Baldassari L.E., Atkins H.L., Bowen J.D., Bredeson C., Carpenter P.A., Corboy J.R., Freedman M.S., Griffith L.M., Lowsky R. (2019). Autologous hematopoietic cell transplantation for treatment-refractory relapsing multiple sclerosis: Position statement from the american society for blood and marrow transplantation. Biol. Blood Marrow Transplant..

[5] Greco R., Labopin M., Badoglio M., Veys P., Silva J.M.F., Abinun M., Gualandi F., Bornhauser M., Ciceri F., Saccardi R. (2019). Allogeneic hsct for autoimmune diseases: A retrospective study from the ebmt adwp, iewp, and pdwp working parties. Front. Immunol..

[6] Passweg J.R., Baldomero H., Chabannon C., Corbacioglu S., de la Cámara R., Dolstra H., Glass B., Greco R., Mohty M., Neven B. (2022). Impact of the sars-cov-2 pandemic on hematopoietic cell transplantation and cellular therapies in europe 2020: A report from the ebmt activity survey. Bone Marrow Transplant..

[7] Greco R. (2022). Personal communication.

[8] Ikehara S., Good R.A., Nakamura T., Sekita K.I., Inoue S., Oo M.M., Muso E., Ogawa K., Hamashima Y. (1985). Rationale for bone marrow transplantation in the treatment of autoimmune diseases. Proc. Natl. Acad. Sci. USA.

[9] Van Bekkum D.W., Bohre E., Houben P., Knaan-Shanzer S. (1989). Regression of adjuvant-induced arthritis in rats following bone marrow transplantation. Proc. Natl. Acad. Sci. USA.

[10] van Bekkum D.W. (1998). New opportunities for the treatment of severe autoimmune diseases: Bone marrow transplantation. Clin. Immunol. Immunopathol..

[11] Knaan-Shanzer S., Houben P., Kinwel-Bohré E.P., van Bekkum D.W. (1991). Remission induction of adjuvant arthritis in rats by total body irradiation and autologous bone marrow transplantation. Bone Marrow Transplant..

[12] Karussis D., Slavin S., Lehmann D., Mizrachi-Koll R., Abramsky O., Ben-Nun A. (1992). Prevention of experimental autoimmune encephalomyelitis and induction of tolerance with acute immunosuppression followed by syngeneic bone marrow transplantation. J. Immunol..

[13] van Gelder M., Kinwel-Bohre E.P., van Bekkum D.W. (1993). Treatment of experimental allergic encephalomyelitis in rats with total body irradiation and syngeneic bmt. Bone Marrow Transplant..

[14] Pestronk A., Drachman D.B., Teoh R., Adams R.N. (1983). Combined short-term immunotherapy for experimental autoimmune myasthenia gravis. Ann. Neurol..

[15] Alexander T., Thiel A., Rosen O., Massenkeil G., Sattler A., Kohler S., Mei H., Radtke H., Gromnica-Ihle E., Burmester G.R. (2009). Depletion of autoreactive immunologic memory followed by autologous hematopoietic stem cell transplantation in patients with refractory sle induces long-term remission through de novo generation of a juvenile and tolerant immune system. Blood.

[16] Brinkman D.M., de Kleer I.M., Cate R., van Rossum M.A., Bekkering W.P., Fasth A., van Tol M.J., Kuis W., Wulffraat N.M., Vossen J.M. (2007). Autologous stem cell transplantation in children with severe progressive systemic or polyarticular juvenile idiopathic arthritis: Long-term follow-up of a prospective clinical trial. Arthritis Rheum..

[17] Farge D., Arruda L.C., Brigant F., Clave E., Douay C., Marjanovic Z., Deligny C., Maki G., Gluckman E., Toubert A. (2017). Long-term immune reconstitution and t cell repertoire analysis after autologous hematopoietic stem cell transplantation in systemic sclerosis patients. J. Hematol. Oncol..

[18] Harris K.M., Lim N., Lindau P., Robins H., Griffith L.M., Nash R.A., Turka L.A., Muraro P.A. (2020). Extensive intrathecal t cell renewal following hematopoietic transplantation for multiple sclerosis. JCI Insight.

[19] Muraro P.A., Douek D.C., Packer A., Chung K., Guenaga F.J., Cassiani-Ingoni R., Campbell C., Memon S., Nagle J.W., Hakim F.T. (2005). Thymic output generates a new and diverse tcr repertoire after autologous stem cell transplantation in multiple sclerosis patients. J. Exp. Med..

[20] Muraro P.A., Robins H., Malhotra S., Howell M., Phippard D., Desmarais C., de Paula Alves Sousa A., Griffith L.M., Lim N., Nash R.A. (2014). T cell repertoire following autologous stem cell transplantation for multiple sclerosis. J. Clin. Investig..

[21] Abrahamsson S.V., Angelini D.F., Dubinsky A.N., Morel E., Oh U., Jones J.L., Carassiti D., Reynolds R., Salvetti M., Calabresi P.A. (2013). Non-myeloablative autologous haematopoietic stem cell transplantation expands regulatory cells and depletes il-17 producing mucosal-associated invariant t cells in multiple sclerosis. Brain.

[22] Arruda L.C., Lorenzi J.C., Sousa A.P., Zanette D.L., Palma P.V., Panepucci R.A., Brum D.S., Barreira A.A., Covas D.T., Simoes B.P. (2015). Autologous hematopoietic sct normalizes mir-16, -155 and -142-3p expression in multiple sclerosis patients. Bone Marrow Transplant..

[23] Delemarre E.M., van den Broek T., Mijnheer G., Meerding J., Wehrens E.J., Olek S., Boes M., van Herwijnen M.J., Broere F., van Royen A. (2016). Autologous stem cell transplantation aids autoimmune patients by functional renewal and tcr diversification of regulatory t cells. Blood.

[24] de Kleer I., Vastert B., Klein M., Teklenburg G., Arkesteijn G., Yung G.P., Albani S., Kuis W., Wulffraat N., Prakken B. (2006). Autologous stem cell transplantation for autoimmunity induces immunologic self-tolerance by reprogramming autoreactive t cells and restoring the cd4+cd25+ immune regulatory network. Blood.

[25] Burt R.K., Balabanov R., Han X., Burns C., Gastala J., Jovanovic B., Helenowski I., Jitprapaikulsan J., Fryer J.P., Pittock S.J. (2019). Autologous nonmyeloablative hematopoietic stem cell transplantation for neuromyelitis optica. Neurology.

[26] Karnell F.G., Lin D., Motley S., Duhen T., Lim N., Campbell D.J., Turka L.A., Maecker H.T., Harris K.M. (2017). Reconstitution of immune cell populations in multiple sclerosis patients after autologous stem cell transplantation. Clin. Exp. Immunol..

[27] Kröger N., Zagrivnaja M., Schwartz S., Badbaran A., Zabelina T., Lioznov M., Ayuk F., Zander A., Fehse B. (2006). Kinetics of plasma-cell chimerism after allogeneic stem cell transplantation by highly sensitive real-time pcr based on sequence polymorphism and its value to quantify minimal residual disease in patients with multiple myeloma. Exp. Hematol..

[28] Suzuki I., Milner E.C., Glas A.M., Hufnagle W.O., Rao S.P., Pfister L., Nottenburg C. (1996). Immunoglobulin heavy chain variable region gene usage in bone marrow transplant recipients: Lack of somatic mutation indicates a maturational arrest. Blood.

[29] Avanzini M.A., Locatelli F., Santos C.D., Maccario R., Lenta E., Oliveri M., Giebel S., De Stefano P., Rossi F., Giorgiani G. (2005). B lymphocyte reconstitution after hematopoietic stem cell transplantation: Functional immaturity and slow recovery of memory cd27+ b cells. Exp. Hematol..

[30] Daikeler T., Labopin M., Di Gioia M., Abinun M., Alexander T., Miniati I., Gualandi F., Fassas A., Martin T., Schwarze C.P. (2011). Secondary autoimmune diseases occurring after hsct for an autoimmune disease: A retrospective study of the ebmt autoimmune disease working party. Blood.

[31] Loh Y., Oyama Y., Statkute L., Quigley K., Yaung K., Gonda E., Barr W., Jovanovic B., Craig R., Stefoski D. (2007). Development of a secondary autoimmune disorder after hematopoietic stem cell transplantation for autoimmune diseases: Role of conditioning regimen used. Blood.

[32] Massarotti C., Sbragia E., Boffa G., Vercelli C., Zimatore G.B., Cottone S., Frau J., Raiola A., Varaldo R., Mancardi G. (2021). Menstrual cycle resumption and female fertility after autologous hematopoietic stem cell transplantation for multiple sclerosis. Mult. Scler. J..

[33] Massenkeil G., Alexander T., Rosen O., Dorken B., Burmester G., Radbruch A., Hiepe F., Arnold R. (2016). Long-term follow-up of fertility and pregnancy in autoimmune diseases after autologous haematopoietic stem cell transplantation. Rheumatol. Int..

[34] Snarski E., Snowden J.A., Oliveira M.C., Simoes B., Badoglio M., Carlson K., Burman J., Moore J., Rovira M., Clark R.E. (2015). Onset and outcome of pregnancy after autologous haematopoietic sct (ahsct) for autoimmune diseases: A retrospective study of the ebmt autoimmune diseases working party (adwp). Bone Marrow Transplant..

[35] Muraro P.A., Pasquini M., Atkins H.L., Bowen J.D., Farge D., Fassas A., Freedman M.S., Georges G.E., Gualandi F., Hamerschlak N. (2017). Long-term outcomes after autologous hematopoietic stem cell transplantation for multiple sclerosis. JAMA Neurol..

[36] Snowden J.A., Badoglio M., Labopin M., Giebel S., McGrath E., Marjanovic Z., Burman J., Moore J., Rovira M., Wulffraat N.M. (2017). Evolution, trends, outcomes, and economics of hematopoietic stem cell transplantation in severe autoimmune diseases. Blood Adv..

[37] Muraro P.A., Martin R., Mancardi G.L., Nicholas R., Sormani M.P., Saccardi R. (2017). Autologous haematopoietic stem cell transplantation for treatment of multiple sclerosis. Nat. Rev. Neurol..

[38] Sormani M.P., Muraro P.A., Schiavetti I., Signori A., Laroni A., Saccardi R., Mancardi G.L. (2017). Autologous hematopoietic stem cell transplantation in multiple sclerosis: A meta-analysis. Neurology.

[39] Peng F., Qiu W., Li J., Hu X., Huang R., Lin D., Bao J., Jiang Y., Bian L. (2010). A preliminary result of treatment of neuromyelitis optica with autologous peripheral hematopoietic stem cell transplantation. Neurologist.

[40] Matiello M., Pittock S.J., Porrata L., Weinshenker B.G. (2011). Failure of autologous hematopoietic stem cell transplantation to prevent relapse of neuromyelitis optica. Arch. Neurol..

[41] Aouad P., Li J., Arthur C., Burt R., Fernando S., Parratt J. (2015). Resolution of aquaporin-4 antibodies in a woman with neuromyelitis optica treated with human autologous stem cell transplant. J. Clin. Neurosci..

[42] Hoay K.Y., Ratnagopal P. (2018). Autologous hematopoietic stem cell transplantation for the treatment of neuromyelitis optica in singapore. Acta Neurol. Taiwan.

[43] Greco R., Bondanza A., Oliveira M.C., Badoglio M., Burman J., Piehl F., Hagglund H., Krasulova E., Simoes B.P., Carlson K. (2015). Autologous hematopoietic stem cell transplantation in neuromyelitis optica: A registry study of the ebmt autoimmune diseases working party. Mult. Scler..

[44] Burton J.M., Duggan P., Costello F., Metz L., Storek J. (2021). A pilot trial of autologous hematopoietic stem cell transplant in neuromyelitis optic spectrum disorder. Mult. Scler. Relat. Disord..

[45] Ceglie G., Papetti L., Valeriani M., Merli P. (2020). Hematopoietic stem cell transplantation in neuromyelitis optica-spectrum disorders (nmo-sd): State-of-the-art and future perspectives. Int. J. Mol. Sci..

[46] Nabizadeh F., Masrouri S., Sharifkazemi H., Azami M., Nikfarjam M., Moghadasi A.N. (2022). Autologous hematopoietic stem cell transplantation in neuromyelitis optica spectrum disorder: A systematic review and meta-analysis. J. Clin. Neurosci..

[47] Hadavi S., Noyce A.J., Leslie R.D., Giovannoni G. (2011). Stiff person syndrome. Pract. Neurol..

[48] Martinez-Hernandez E., Arino H., McKeon A., Iizuka T., Titulaer M.J., Simabukuro M.M., Lancaster E., Petit-Pedrol M., Planaguma J., Blanco Y. (2016). Clinical and immunologic investigations in patients with stiff-person spectrum disorder. JAMA Neurol..

[49] Newsome S.D., Johnson T. (2022). Stiff person syndrome spectrum disorders; more than meets the eye. J. Neuroimmunol..

[50] Baizabal-Carvallo J.F., Jankovic J. (2015). Stiff-person syndrome: Insights into a complex autoimmune disorder. J. Neurol. Neurosurg. Psychiatry.

[51] Dalakas M.C., Fujii M., Li M., Lutfi B., Kyhos J., McElroy B. (2001). High-dose intravenous immune globulin for stiff-person syndrome. N. Engl. J. Med..

[52] Baker M.R., Das M., Isaacs J., Fawcett P.R., Bates D. (2005). Treatment of stiff person syndrome with rituximab. J. Neurol. Neurosurg. Psychiatry.

[53] McKeon A., Robinson M.T., McEvoy K.M., Matsumoto J.Y., Lennon V.A., Ahlskog J.E., Pittock S.J. (2012). Stiff-man syndrome and variants: Clinical course, treatments, and outcomes. Arch. Neurol..

[54] Sanders S., Bredeson C., Pringle C.E., Martin L., Allan D., Bence-Bruckler I., Hamelin L., Hopkins H.S., Sabloff M., Sheppard D. (2014). Autologous stem cell transplantation for stiff person syndrome: Two cases from the ottawa blood and marrow transplant program. JAMA Neurol..

[55] Georges G.E., Bowen J.D., Pearlman M., Wundes A., von Geldern G., Kraft G.H., Weiss M.D., McLaughlin B., Sytsma J., Nash R. (2018). Autologous hematopoietic stem cell transplantation may be highly effective treatment for severe stiff person syndrome. Biol. Blood Marrow Transplant..

[56] Kass-Iliyya L., Snowden J.A., Thorpe A., Jessop H., Chantry A.D., Sarrigiannis P.G., Hadjivassiliou M., Sharrack B. (2021). Autologous haematopoietic stem cell transplantation for refractory stiff-person syndrome: The uk experience. J. Neurol..

[57] Burt R.K., Balabanov R., Han X., Quigley K., Arnautovic I., Helenowski I., Rose J., Siddique T. (2021). Autologous hematopoietic stem cell transplantation for stiff-person spectrum disorder: A clinical trial. Neurology.

[58] Dalakas M.C. (2021). Limited benefits halt enrollment in hematopoietic stem cell transplantation trial for stiff-person syndrome: Should there be more to come?. Neurology.

[59] Lehmann H.C., Burke D., Kuwabara S. (2019). Chronic inflammatory demyelinating polyneuropathy: Update on diagnosis, immunopathogenesis and treatment. J. Neurol. Neurosurg. Psychiatry.

[60] Vermeulen M., Van Oers M. (2002). Successful autologous stem cell transplantation in a patient with chronic inflammatory demyelinating polyneuropathy. J. Neurol. Neurosurg. Psychiatry.

[61] Vermeulen M., Van Oers M. (2007). Relapse of chronic inflammatory demyelinating polyneuropathy 5 years after autologous stem cell transplantation. J. Neurol. Neurosurg. Psychiatry.

[62] Mahdi-Rogers M., Kazmi M., Ferner R., Hughes R.A., Renaud S., Steck A.J., Fuhr P., Halter J., Gratwohl A., Tyndall A. (2009). Autologous peripheral blood stem cell transplantation for chronic acquired demyelinating neuropathy. J. Peripher. Nerv. Syst..

[63] Press R., Askmark H., Svenningsson A., Andersen O., Axelson H.W., Strömberg U., Wahlin A., Isaksson C., Johansson J.J., Hägglund H. (2014). Autologous haematopoietic stem cell transplantation: A viable treatment option for cidp. J. Neurol. Neurosurg. Psychiatry.

[64] Ajroud-Driss S., Gozdziak P., Sufit R., Burt R. (2011). Non-myeloablative autologous hematopoietic stem cell transplantation for the treatment of refractory cidp. J. Peripher. Nerv. Syst..

[65] Masson-Roy J., Breiner A., Warman-Chardon J., Pringle C.E., Allan D., Bredeson C., Huebsch L., Kekre N., Kennah M.L. (2021). Martin. Autologous hematopoietic stem cell transplantation for chronic inflammatory demyelinating polyradiculoneuropathy. Can. J. Neurol. Sci..

[66] Burt R.K., Balabanov R., Tavee J., Han X., Sufit R., Ajroud-Driss S., Jovanovic B., Quigley K. (2020). Arnautovic and I. Helenowski. Hematopoietic stem cell transplantation for chronic inflammatory demyelinating polyradiculoneuropathy. J. Neurol..

[67] Allen J., Sufit R., Ajroud-Driss S., Burt R. (2013). Nonmyeloablative autologous hematopoetic stem cell transplant for the treatment of cidp: An interim report. J. Peripher. Nerv. Syst..

[68] Burt R.K., Tappenden P., Balabanov R., Han X., Quigley K., Snowden J.A., Sharrack B. (2021). The cost effectiveness of immunoglobulin vs. Hematopoietic stem cell transplantation for cidp. Front. Neurol..

[69] Gilhus N.E. (2016). Myasthenia gravis. N. Engl. J. Med..

[70] Menon D., Bril V. (2022). Pharmacotherapy of generalized myasthenia gravis with special emphasis on newer biologicals. Drugs.

[71] Bryant A., Atkins H., Pringle C.E., Allan D., Anstee G., Bence-Bruckler I., Hamelin L., Hodgins M., Hopkins H., Huebsch L. (2016). Myasthenia gravis treated with autologous hematopoietic stem cell transplantation. JAMA Neurol..

[72] Sossa Melo C.L., Peña A.M., Salazar L.A., Jiménez S.I., Gómez E.D., Chalela C.M., Ayala-Castillo M., Peña I.M. (2019). Autologous hematopoietic stem cell transplantation in a patient with refractory seropositive myasthenia gravis: A case report. Neuromuscul. Disord..

[73] Håkansson I., Sandstedt A., Lundin F., Askmark H., Pirskanen R., Carlson K., Piehl F., Hägglund H. (2017). Successful autologous haematopoietic stem cell transplantation for refractory myasthenia gravis-a case report. Neuromuscul. Disord..

[74] Strober J., Cowan M.J., Horn B.N. (2009). Allogeneic hematopoietic cell transplantation for refractory myasthenia gravis. Arch. Neurol..

[75] Inan B., Bekircan-Kurt C.E., Demiroğlu H., Göker H., Erdem-Özdamar S., Tan E. (2022). Autologous stem cell transplantation in a patient with refractory anti-musk-positive myasthenia gravis and familial mediterranean fever. Neurol. Sci. Neurophysiol..

[76] Mitsumune S., Manabe Y., Yunoki T., Kono S., Aoyama K., Shinno Y., Narai H., Abe K. (2018). Autologous bone marrow transplantation for polymyositis combined with myasthenia gravis and aplastic anemia: A case report. Case Rep. Neurol..

[77] Clinicaltrials.gov (2022). Hematopoietic Stem Cell Therapy for Patients with Refractory Myasthenia Gravis. https://clinicaltrials.gov/ct2/show/study/NCT00424489.

[78] Findlay A.R., Goyal N.A., Mozaffar T. (2015). An overview of polymyositis and dermatomyositis. Muscle Nerve.

[79] Baron F., Ribbens C., Kaye O., Fillet G., Malaise M., Beguin Y. (2000). Effective treatment of jo-1-associated polymyositis with t-cell-depleted autologous peripheral blood stem cell transplantation. Br. J. Haematol..

[80] Holzer U., van Royen-Kerkhof A., Van Der Torre P., Kuemmerle-Deschner J., Well C., Handgretinger R., Mueller I., Wulffraat N. (2010). Successful autologous stem cell transplantation in two patients with juvenile dermatomyositis. Scand. J. Rheumatol..

[81] Zhu J., Su G., Lai J., Dong B., Kang M., Li S., Zhou Z., Wu F. (2018). Long-term follow-up of autologous hematopoietic stem cell transplantation for refractory juvenile dermatomyositis: A case-series study. Pediatr. Rheumatol..

[82] Chahin N., Selcen D., Engel A.G. (2005). Sporadic late onset nemaline myopathy. Neurology.

[83] Belkhribchia M.R., Tazi I., Louhab N., Kissani N., Mahmal L., Pereon Y. (2017). Autologous stem cell transplantation in a patient with sporadic late-onset nemaline myopathy and monoclonal gammopathy: First moroccan experience. Presse Médicale.

[84] Voermans N.C., Benveniste O., Minnema M.C., Lokhorst H., Lammens M., Meersseman W., Delforge M., Kuntzer T., Novy J., Pabst T. (2014). Sporadic late-onset nemaline myopathy with mgus: Long-term follow-up after melphalan and sct. Neurology.

[85] Hanbali A., Rasheed W., Peedikayil M.C., Boholega S., Alzahrani H.A. (2018). Mitochondrial neurogastrointestinal encephalomyopathy syndrome treated with stem cell transplant: A case series and literature review. Exp. Clin. Transplant..

[86] Halter J.P., Michael W., Schüpbach M., Mandel H., Casali C., Orchard K., Collin M., Valcarcel D., Rovelli A., Filosto M. (2015). Allogeneic haematopoietic stem cell transplantation for mitochondrial neurogastrointestinal encephalomyopathy. Brain.

[87] Hirano M., Marti R., Casali C., Tadesse S., Uldrick T., Fine B., Escolar D., Valentino M., Nishino I., Hesdorffer C. (2006). Allogeneic stem cell transplantation corrects biochemical derangements in mngie. Neurology.

[88] Born A.P., Müller K., Marquart H.V., Heilmann C., Schejbel L., Vissing J. (2010). Myositis in griscelli syndrome type 2 treated with hematopoietic cell transplantation. Neuromuscul. Disord..

[89] Pavlakis P.P. (2020). Rheumatologic disorders and the nervous system. Continuum.

[90] Daikeler T., Kötter I., Tyndall C.B., Apperley J., Attarbaschi A., Guardiola P., Gratwohl A., Jantunen E., Marmont A., Porretto F. (2007). Haematopoietic stem cell transplantation for vasculitis including behcet’s disease and polychondritis: A retrospective analysis of patients recorded in the european bone marrow transplantation and european league against rheumatism databases and a review of the literature. Ann. Rheum. Dis..

[91] De Cata A., Intiso D., Bernal M., Molinaro F., Mazzoccoli G., D’Alessandro V., Greco A., Curci S., Sperandeo M., Frusciante V. (2007). Prolonged remission of neuro-behcet disease following autologous transplantation. Int. J. Immunopathol. Pharmacol..

[92] Marmont A.M., Gualandi F., Piaggio G., Podestà M., van Lint M.T., Bacigalupo A., Nobili F. (2006). Allogeneic bone marrow transplantation (bmt) for refractory behçet’s disease with severe cns involvement. Bone Marrow Transplant..

[93] Statkute L., Oyama Y., Barr W.G., Sufit R., Ho S., Verda L., Loh Y., Yaung K., Quigley K., Burt R.K. (2008). Autologous non-myeloablative haematopoietic stem cell transplantation for refractory systemic vasculitis. Ann. Rheum. Dis..

[94] Burt R.K., Traynor A., Statkute L., Barr W.G., Rosa R., Schroeder J., Verda L., Krosnjar N., Quigley K., Yaung K. (2006). Nonmyeloablative hematopoietic stem cell transplantation for systemic lupus erythematosus. JAMA.

[95] Lehnhardt F.G., Scheid C., Holtik U., Burghaus L., Neveling M., Impekoven P., Rüger A., Hallek M., Jacobs A.H., Rubbert A. (2006). Autologous blood stem cell transplantation in refractory systemic lupus erythematodes with recurrent longitudinal myelitis and cerebral infarction. Lupus.

[96] Trysberg E., Lindgren I., Tarkowski A. (2000). Autologous stem cell transplantation in a case of treatment resistant central nervous system lupus. Ann. Rheum. Dis..

[97] Goklemez S., Hasni S., Hakim F.T., Muraro P.A., Pirsl F., Rose J., Memon S., Fowler D.F., Steinberg S.M., Baker E.H. (2022). Long-term follow-up after lymphodepleting autologous haematopoietic cell transplantation for treatment-resistant systemic lupus erythematosus. Rheumatology.

[98] Lisukov I.A., Sizikova S.A., Kulagin A.D., Kruchkova I.V., Gilevich A.V., Konenkova L.P., Zonova E.V., Chernykh E.R., Leplina O.Y., Sentyakova T.N. (2004). High-dose immunosuppression with autologous stem cell transplantation in severe refractory systemic lupus erythematosus. Lupus.

[99] Froehlich M., Schwaneck E.C., Gernert M., Gadeholt O., Strunz P.-P., Morbach H., Tony H.-P., Schmalzing M. (2020). Autologous stem cell transplantation in common variable immunodeficiency: A case of successful treatment of severe refractory autoimmune encephalitis. Front. Immunol..

[100] Snowden J.A., Sanchez-Ortega I., Corbacioglu S., Basak G.W., Chabannon C., de la Camara R., Dolstra H., Duarte R.F., Glass B., Greco R. (2022). Indications for haematopoietic cell transplantation for haematological diseases, solid tumours and immune disorders: Current practice in europe, 2022. Bone Marrow Transplant..

[101] Burt R.K., Han X., Gozdziak P., Yaung K., Morgan A., Clendenan A.M., Henry J., Calvario M.A., Datta S.K., Helenowski I. (2018). Five year follow-up after autologous peripheral blood hematopoietic stem cell transplantation for refractory, chronic, corticosteroid-dependent systemic lupus erythematosus: Effect of conditioning regimen on outcome. Bone Marrow Transplant..

[102] Greco R., Alexander T., Burman J., Del Papa N., de Vries-Bouwstra J., Farge D., Henes J., Kazmi M., Kirgizov K., Muraro P.A. (2021). Hematopoietic stem cell transplantation for autoimmune diseases in the time of covid-19: Ebmt guidelines and recommendations. Bone Marrow Transplant..

[103] Clinicaltrials.gov Autologous Peripheral Blood Stem Cell Transplant for Neurologic Autoimmune Diseases. https://clinicaltrials.gov/ct2/show/study/NCT00716066?term=hematopoietic+stem+cell&cond=CIDP&draw=2&rank=2.

[104] Clinicaltrials.gov (2022). Stem Cell Transplantation in Idiopathic Inflammatory Myopathy Diseases. https://clinicaltrials.gov/ct2/show/results/NCT00278564?term=hematopoietic+stem+cell&cond=Neurological+Disease&draw=2&rank=5.

